# A one-dimensional extremely covalent material: monatomic carbon linear chain

**DOI:** 10.1186/1556-276X-6-577

**Published:** 2011-10-31

**Authors:** Yaozhong Zhang, Yanjie Su, Liang Wang, Eric Siu-Wai Kong, XiaoShuang Chen, Yafei Zhang

**Affiliations:** 1 Key Laboratory for Thin Film and Microfabrication of the Ministry of Education, Research Institute of Micro/Nano Science and Technology, Shanghai Jiao Tong University, Shanghai 200240, China; 2 National Laboratory for Infrared Physics, Shanghai Institute of Technical Physics, Chinese Academy of Sciences, Shanghai 200083, China

**Keywords:** carbon atomic chains, Young's modulus, heat capacity, single-walled carbon nanotubes

## Abstract

Polyyne and cumulene of infinite length as the typical covalent one-dimensional (1D) monatomic linear chains of carbon have been demonstrated to be metallic and semiconductor (Eg = 1.859 eV), respectively, by first-principles calculations. Comparing with single-walled carbon nanotubes, the densities are evidently low and the thermodynamic properties are similar below room temperature but much different at the high temperature range. Polyyne possesses a Young's modulus as high as 1.304 TPa, which means it is even much stiffer than carbon nanotubes and to be the superlative strong 1D material along the axial direction. The Young's modulus of cumulene is estimated to be 760.78 GPa. In addition, polyyne is predicted to be as a one-dimensional electronic material with very high mobility.

## Introduction

Carbon nanomaterials possess different mechanical and electronic properties when the hybridization of carbon atoms changes from sp, sp^2 ^to sp^3^. As quasi one-dimensional carbon nanostructures, carbon nanotubes [1], composed of sp^2^/sp^3^-hybridized carbon atoms, possess outstanding mechanical, optical, and electronic properties, offering carbon nanotubes many promising applications as composite materials and sensors, as well as technology areas such as nanoelectronics. Graphene [2], a one-atom-thick two-dimensional sheet of sp^2^-hybridized carbon atoms, has also attracted increasing attentions due to its excellent thermal conductivity, mechanical stiffness, and extraordinary electronic transport properties, rendering graphene potentially valuable for applications in nanoelectronics, solar cell window layers, and composite materials [3]. Among all carbon nanostructures, carbon atomic chain, comprised of monatomic linear chains of carbon atoms, is a truly one-dimensional nanomaterial and has been predicted to have extremely outstanding properties for various applications [4-6]. Recently, Zhao et al reported the experimental fabrication of monatomic carbon linear chain which was shown to be inside double-walled carbon nanotubes of 0.7 nm in diameter [4]. Three characteristic Raman shift peaks were observed in the range of 1,790 to 1,860 cm^-1^. Mikhailovskij et al. reported fabrication of free-standing short monatomic carbon linear chains using a developed experimental high-field technique [7]. However, desirable quantity of free-standing polyyne and cumulene samples for experimental characterization has not gotten until now.

Among various possible covalent one-dimensional (1D) monatomic linear chains of carbon, cumulene of infinite length (···C ≡ C - C···) constructed with alternate sp conjugated band and sp^3 ^conjugated bond chain and polyyne of infinite length (···C = C = C···) with sp^2 ^conjugated bond chain are typical rigid covalent period conjugated bond chains. Polyynes are found in interstellar molecular clouds where hydrogen is scarce. The longest reported (synthetic) polyyne to date contains 22 acetylenic units and is end-capped with triisopropylsilyl groups [8]. The polyyne of infinite length is the elusive compound called carbyne; linear acetylenic carbon is one of the carbon allotropes [9]. To date, the difference of physical properties in these two types of carbon atomic chains has not been reported. However, there is no effective method reported in the literature to synthesize and characterize free-standing carbon atomic chains. In this investigation, theoretical study of carbon atomic chains properties has been conducted. Two kinds of carbon atomic chains have been investigated by first-principles calculations. Periodic carbon atomic chain structure has been created and geometry optimized in order to build a stable structure. Thermodynamic, mechanical, and electronic properties of carbon atomic chains have been calculated, and the results of physical properties are discussed.

## Calculations

First-principles calculations have been performed to investigate the physical properties of carbon atomic chains by the pseudo-potential plane wave method [10] with the generalized gradient approximation [11] of Perdew-Burke-Ernzerhof for the exchange-correlation potential [12].

For all structures, the Monkhorst-Pack scheme is used in the Brillouin zone with 2 × 2 × 10 for all the geometry optimization and total energy calculations [13]. For all relaxation calculations, the volume of cell is fixed and the structure considered are fully relaxed to accuracy where the self-consistent field was done with a convergence criterion of 2 × 10^-5 ^eV/atom.

Since experiment data on physical properties of single-walled carbon nanotubes (SWNTs) have been extensively reported [14], the model of a (5,5) SWNT is chosen as a reference for first-principles calculation: the physical data are compared in order to confirm the accuracy of calculation method and the feasibility of carbon atomic chain.

**Figure 1:**
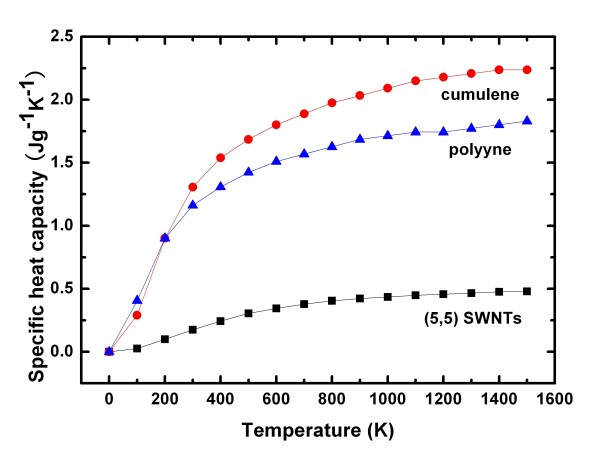
**Geometrical structure model**. (**a**) (5,5) SWNTs, (**b**) cumulene, and (**c**) polyyne.

With the reference of the distance between two neighbor layers in graphite is 3.42 Å, and linear monatomic carbon linear chain was shown to be inside double-walled carbon nanotubes of 0.7 nm in diameter, therefore, the smallest distance can be chosen as 3.42 Å between two free carbon atomic chains. The geometrical structure model of (5,5) SWNT, cumulene, and polyyne are shown in Figure 1.

The calculated geometrical model of (5,5) SWNT contains 80 carbon atoms, as shown in Figure 1a, with periodic border condition. Structural optimization has been established using a bond length of 1.42 Å, which is slight shorter than that in graphene inducing from the surface curving of the SWNT. The model of cumulene as shown in Figure 1b is built with 12 carbon atoms with alternate sp conjugated band and sp^3 ^bonds and periodic border condition. The length of sp conjugated bond is 0.1264 nm and that of sp^3 ^bond is 0.1476 nm. Polyyne is in the same set as cumulene, as shown in Figure 1c with 12 carbon atoms, and there are only sp^2 ^conjugated bond of length up to 0.1404 nm with periodic border condition.

## Results and discussion

The calculated data of (5,5) SWNT, cumulene, and polyyne are listed in Table 1 in which diamond and graphene are included for comparing. At first, the structure stability of cumulene and polyyne is demonstrated to be lower than SWNT because their Gibbs free energy is more minus than that of (5,5) SWNT. Secondly, SWNT has been known as a super light one-dimensional material. However, cumulene and polyyne have even much lower density for potential applications as typical one-dimensional carbon materials.

**Table 1:** Calculated data of (5,5) SWNT, cumulene, and polyyne

	Diamond	Graphene	(5,5) SWNT	Cumulene	Polyyne
Bond length (nm)	0.154	0.142	0.1419	0.1264/0.1476	0.1404
Gibbs free energy (KJ/mol)	-	-	-4,502.4	-19,678.2	-14,777.9
Density (g/cm^3^)	3.52	2.23	1.58	1.24	1.28
Young's modulus (TPa)	1.050	-	0.805	0.760	1.304
Specific heat capacity (J/g·K) (300 K)	-	-	0.174	1.307	1.162
Bandgap (eV)	5.450	0.000	0.000	1.859	0.000
Electron mobility (cm^2^/V·s)	2200	200,000	100,000	Very high?	Highest?

It has been reported that the experimental Young's modulus of (5,5) SWNT is about 0.3 to 0.6 TPa with zero bandgap [15,16]. The calculated Young's modulus of SWNTs in this investigation is 805.12 GPa, which is quite close to the experiment data, demonstrating the successful application of the first-principles theory to calculate the mechanical properties of carbon nanoscale structures. The mechanical properties and bandgap energy of (5,5) SWNTs and carbon atomic chains are listed in Table 1.

The calculated Young's modulus of polyyne is as high as 1,304.19 GPa, which is much larger than that of (5,5) SWNTs, cumulene (760.78 GPa), or any known materials. The high Young's modulus of polyyne can be attributed to the high strength of sp^2 ^conjugated bonds. We note, however, that in the SWNT structure, the carbon bonds may be considered as a hybrid sp*^x ^*(2 <*x *< 3) bond, which is much weaker than sp^2 ^conjugated bond. In the cumulene structure, there exists the single sp^3 ^bond, which is even weaker than the sp*^x ^*(2 <*x *< 3) bond in SWNT structure. Therefore, we conclude that polyyne possesses the highest strength along its axial compared to any other materials.

The calculation results show that cumulene is a semiconductor (Eg = 1.859 eV), but polyyne is metallic. As we know, the electronic conductivity of carbon structures comes from enlarged moving range of electrons through sp^2 ^conjugated bonds, which decrease the system energy level and result in the effect of the electron mobility increasing significantly [17-20]. Because of a single sp^3 ^carbon bond in the cumulene structure, the electronic conductivity is much lower than that of polyyne, and the bandgap energy of cumulene widens and renders cumulene to be a semiconductor.

**Figure 2:**
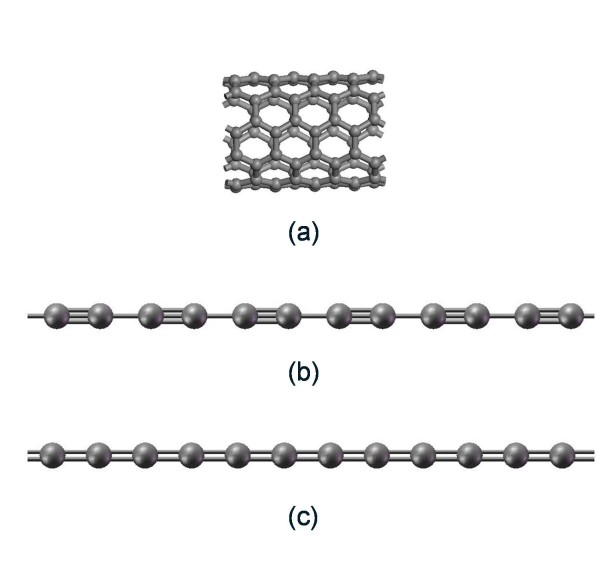
**Specific heat capacity sketch of cumulene, polyyne and (5,5) SWNT**.

The specific heat capacity chart of cumulene in Figure 2 shows that heat capacity increases with increasing temperature. According to the thermal equation:

(1) K=c⋅m⋅X

where *K *is the coefficient of heat conductivity, *c *is specific heat capacity, *m *is mass, and *X *replaces other parameters. Specific heat capacity is proportional to heat capacity; therefore, the coefficient of heat conductivity is proportional to heat capacity [21]. It can be noticed that the heat conductivity coefficient of cumulene increases with rising temperature.

SWNT is a one-dimensional super low-density material, with very high Young's modulus. In this investigation, the heat capacity chart is calculated and shows a trend rises with increasing temperature gradually, as shown in Figure 2. Comparing at 300 K, the specific heat capacities of cumulene, polyyne, and (5,5) SWNT are about 1.307, 1.162, and 0.174 J g^-1 ^K^-1^, respectively. The specific heat capacity of (5,5) SWNT is about an order smaller than that of the cumulene and polyyne. Meanwhile, the increasing rate of the specific heat capacity of (5,5) SWNT is slower grading as the temperature increases from 300 K. These results indicate that the thermodynamic properties of cumulene and polyyne are different obviously from that of SWNTs. SWNT has been known as a super one-dimensional material with very high Young's modulus and low density for application in many fields. However, cumulene and polyyne have even much lower density and extremely high Young's modulus for some potential applications.

On the other hand, low-dimensional carbon materials, such as carbon nanotubes and graphene, have been reported to have very high mobility values of 100,000 cm^2^/V·s [22] and 200,000 cm^2^/V·s [23] at room temperature, respectively. The reason for such high mobility is related to the carbon structure of sp*^x ^*conjugation. For example, graphite with similar carbon structure is a good conductor of electricity. The mobility of charges in graphite is as high as 20,000 cm^2^/V·s at room temperature. Graphite is also strong, lightweight, and an excellent conductor of heat. The mobilities in nanotubes and graphenes turn out to be even higher than in graphite because low-dimensional carbon structures of sp*^x ^*conjugation behave as high-mobility electron gases. Another reason may lie in the quantum confinement nature of the low-dimensional carbon structure, which is harder to scatter electrons in low-dimensional confined structure. According to the typical structure of polyyne, it has the most favorable quantum confined carbon structure of sp*^x ^*conjugation for possessing a possible super high electron mobility and super conductivity as well. However, cumulene may have a relatively lower electron mobility due to its sp conjugated band and sp^3 ^bond alternate chain structure. Electrons can only go forward or backward but not sideways. Metallic carbon atomic chains can behave as one-dimensional electron gases, having outstanding properties in bandgap energy as well as Young's modulus. Indeed, the results of this investigation suggest that monatomic carbon linear chains, cumulene and polyyne, have promising potential application in many fields.

## Conclusions

First-principles calculations have been performed to investigate the properties of covalent one-dimensional (1D) monatomic linear chains of carbon. Polyyne and cumulene of infinite length, as the two typical chains, have been studied with SWNT, graphene, and diamond to reveal the fundamental and technologically interesting features. The Young's modulus of polyyne has been demonstrated to be high as 1.304 TPa, which is even much higher than that of (5,5) SWNT (0.805 TPa) and diamond (1.050 TPa). This means that polyyne is an extremely strong 1D material along the axial direction. Specific heat capacity analysis shows that the thermodynamic properties of cumulene and polyyne are different obviously from those of SWNTs and that cumulene and polyyne have much larger specific heat capacity. According to the typical electronic structure of polyyne, a super high electron mobility is predicted to exist in the most favorable quantum confined electron gas structure in 1D. Cumulenes as a semiconductor (Eg ≈ 1.859 eV) and polyynes as a metallic monatomic chains are distinguished from other organic chains by their rigidity, which makes them promising for applications in nanotechnology.

## Competing interests

The authors declare that they have no competing interests.

## Authors' contributions

YZ carried out the study and drafted the manuscript. YS participated in the sequence alignment. LW participated in the design of the study and sequence alignment. ESK participated in the sequence alignment. XC participated in the sequence alignment. YZ participated in the design of the study and sequence alignment.
